# Inhibition of CD83 Alleviates Systemic Inflammation in Herpes Simplex Virus Type 1-Induced Behçet's Disease Model Mouse

**DOI:** 10.1155/2019/5761392

**Published:** 2019-09-09

**Authors:** S. M. Shamsul Islam, Hae-Ok Byun, Bunsoon Choi, Seonghyang Sohn

**Affiliations:** ^1^Department of Biomedical Science, Ajou University School of Medicine, Suwon 16499, Republic of Korea; ^2^Department of Microbiology, Ajou University School of Medicine, Suwon 16499, Republic of Korea; ^3^Institute for Medical Sciences, Ajou University School of Medicine, Suwon 16499, Republic of Korea

## Abstract

Behçet's disease (BD) is an autoinflammatory disease that can lead to life- and sight-threating complications. Dendritic cells (DCs) are the most potent antigen-presenting cells that can regulate multiple inflammatory pathways. The objective of this study was to investigate the association of the DC stimulatory molecule CD83 with BD. Frequencies of costimulatory molecules expressing DCs in peripheral blood leukocytes (PBL) were measured by flow cytometry (FACS). The severity of symptoms in HSV-1-induced BD symptomatic mice was also assessed. Frequencies of CD83-positive cells were significantly increased in mice exhibiting BD symptoms, compared to those in asymptomatic mice. Abatacept, a CD80/86 blocker, significantly decreased the frequencies of CD83-positive cells in a time- and dose-dependent manner. BD symptomatic mice treated with Abatacept showed gradual reduction in the severity score of symptoms. Intraperitoneal injection of CD83 siRNA significantly reduced the frequencies of CD83-positive cells in PBL and peritoneal macrophages. After CD83 siRNA injection, BD symptoms of mice were improved and disease severity was decreased. Discontinuation of CD83 siRNA deteriorated symptoms while readministration of CD83 siRNA again improved BD symptoms of mice. These results clearly indicate the involvement of CD83-expressing cells in the inflammatory symptoms of BD. Therefore, CD83 might be useful as a therapeutic target for BD.

## 1. Introduction

Behçet's disease (BD) is a multisystemic autoinflammatory disease with inflammatory lesion as its main clinical feature that can affect the skin, joints, eye, intestinal tract, genital area, and nervous system. The exact etiology of BD is currently unclear. However, several factors including environmental, genetic, infectious, and/or immunologic dysregulation have been suggested as possible triggering factors. Herpes simplex virus (HSV) is considered as one of the triggering factors in BD. HSV viral DNA particles have been identified in ocular fluids [1], peripheral blood leucocytes [2], saliva [3], and skin lesions [4], of BD patients. Serum anti-HSV-1 antibodies [2] have also been identified in BD patients. HSV-1-induced model mice show similar clinical manifestations, including genital ulcer, oral ulcer, skin lesions, eye lesions, arthritis, and intestinal ulcers [5]. When evaluated in immune modulatory experiments, HSV-1-induced model mice are very similar to those of human BD disease patterns [6].

Dendritic cells (DCs) are the most potent antigen-presenting cells (APCs) that can effectively connect innate and adaptive immune systems. Due to its unique ability to induce the activation and differentiation of T lymphocytes, many investigators focus on DC-mediated immune response. DCs are involved in several autoimmune diseases, such as inflammatory bowel disease (IBD) [7], rheumatoid arthritis (RA) [8], uveitis [9], and Crohn's disease (CD) [10]. Upon antigen capture, DCs undergo a process of maturation. Mature DCs then acquire the ability to differentiate naïve T cells, B cells, and NK cells. They also express cytokines [11]. During maturation, DCs accumulate peptides and upregulate expression levels of the major histocompatibility complex (MHC) and costimulatory molecules such as CD40, CD80, CD83, and CD86 [12]. Among costimulatory molecules, CD83 plays an important role in immune response besides its function as an activation marker [13]. HSV-1-infected DCs can lead to degradation of CD83 within 6 to 8 hours after infection [14]. They also lead to inhibition of the CD83 mRNA transport, thus significantly inhibiting DC-mediated T lymphocyte activation [15]. CD83 upregulation and selective expression, together with CD80 and CD86, suggest an important role of CD83 in immune response [16]. CD83 is a membrane integral protein [17], and soluble CD83 (sCD83) is produced by the release of cell surface CD83 molecules [18]. Elevated levels of sCD83 have been found in plasma and synovial fluids of RA patients [19, 20] and in those with hematological malignancies [21]. Although the functions of CD83 ligands (CD83L) remain controversial, it is believed that when they are stimulated with CD3 and CD28, activated T cells can express CD83L, suggesting that CD83L might function in immune response when T cells are activated in the presence of the costimulatory signal provided by CD83 APCs [22]. The specific role of CD83 in the regulation of immune response is not yet well known. However, the manipulation of the CD83 pathway has been proposed to develop therapeutics for the treatment of inflammation and autoimmune diseases [18]. Blocking CD83 function or its ligand has not yet been demonstrated in BD. Therefore, the purpose of this study was to determine whether blocking CD83 function could affect BD symptoms in a mouse model.

## 2. Materials and Methods

### 2.1. Animal Experiment

Institute of Cancer Research (ICR) (CD1) mice at 4 to 5 weeks old were infected with HSV type 1 (1 × 10^6^ plaque-forming unit (pfu)/mL, F strain) grown in Vero cells as previously described [5]. Virus inoculation was performed twice with a 10-day interval followed by 16 weeks of observation. Mice were bred in temperature- and light-controlled conventional rooms (20-22°C, 12 h light/dark cycle). These mice had *ad libitum* access to food and water. During the experimental period, animals were closely observed and photographed. Animals were handled in accordance with a protocol approved by the Institutional Animal Care and Use Committee of Ajou University (approval number: AMC-2018-0017).

### 2.2. BD Symptomatic Mouse Induced by HSV-1

Virus inoculation was performed using the published procedures [5]. Briefly, earlobes of mice were scratched with a needle and inoculated with 20 *μ*L of 1 × 10^6^ pfu/mL of HSV-1 (F strain) that had been grown in Vero cells. Virus inoculation was performed twice with a 10-day interval. For virus inoculation, mice were euthanized by intramuscular injection in the hind leg with Ketamine/Xylazine cocktail (15 mg/kg Ketamine and 10 mg/kg Xylazine). Several symptoms were observed in mice after HSV inoculation. The incidence of BD was 15% of HSV-inoculated mice, including oral ulcers, genital ulcers, erythema, skin pustules, skin ulcers, arthritis, diarrhea, red eye, loss of balance, and facial swelling. Oral, genital, skin ulcers, and eye symptoms were classified as major symptoms while arthritis, intestinal ulceration, and neurological involvement were considered as minor symptoms. Mice with one or more major symptoms and one or more minor symptoms were classified as having BD. Each symptom score was one. The sum of the scores of different symptoms was used to determine the severity of BD using BD current activity from 2006 prepared by the International Society for Behçet's Disease (http://medhealth.leeds.ac.uk/download/910/behcetsdiseaseactivityform). Loss of symptoms or a reduction in lesion size of more than 20% was an indicator of BD improvement. The control group was inoculated with HSV. Asymptomatic healthy mice were used as BD normal (BDN) as previously described [5].

### 2.3. Medication to the Mice

To normal mice, 1 or 2 mg Abatacept per day was administered for 3 consecutive days via intraperitoneal injection. To BD mice, 2 mg Abatacept per mouse for 3 times with 3-day intervals was applied. As a control group, PBS was treated to normal or BD mice. CD83 siRNA was mixed with jetPEI transfection reagent (Polyplus-transfection, Illkirch-Graffenstaden, France) and used for *in vivo* transfection. For siRNA application to mice, 0.5 or 1.0 *μ*mol of CD83 siRNA was dissolved in 200 *μ*L of 5% glucose solution, mixed with transfection reagent jetPEI, and intraperitoneally injected into normal or BD mice for 4 times with 3-day intervals. As a control, scramble siRNA was applied to normal and BD mice following the same procedure as CD83 siRNA injection. At 2 h after the final injection, mice were sacrificed. Leukocytes isolated from peripheral blood (PBL) and macrophages from the peritoneal cavity were then isolated for further analysis.

### 2.4. Preparation of siRNA

CD83 siRNA oligonucleotides with the following sense and antisense sequences were synthesized by Integrated DNA Technologies (Coralville, IA, USA). Synthesized sequences of CD83 siRNA were as follows: 5′-GUGCUUUUCAGUCAUCUACAAGCTA-3′ and 3′-CUCACGAAAAGUCAGUAGAUGUUCGAU-5′. For injection into mice, CD83 siRNA was mixed with transfection reagent [23].

### 2.5. Generation of Mouse Bone Marrow-Derived DCs

Bone marrow-derived dendritic cells (BMDCs) were obtained from femurs of mice, and red blood cells (RBC) were treated with ACK solution for RBC lysis. These cells were cultured in RPMI media (Gibco, Grand Island, NY, USA) supplemented with 10% fetal bovine serum, 2 ng/mL recombinant mouse IL-4 (ProSpec, NJ, USA), and 20 ng/mL recombinant mouse GM-CSF (ProSpec, NJ, USA). The culture medium was changed at 3 and 6 days after culture. New medium and cytokines (rmIL-4 and rmGM-CSF) were added after rinsing cells. Cells were harvested for experiments on day 9.

### 2.6. siRNA Transfection

Bone marrow-derived cells (2.5 × 10^5^/well) were seeded into 6-well plates in 2 mL of culture medium and treated with cytokines (rmIL-4 and rmGM-CSF). siRNA transfections were performed using jetPEI reagent according to the instructions of the manufacturer. Cells were transfected with 100 ng/well of CD83 siRNA (Integrated DNA Technologies, CA, USA) and scramble siRNA (Bioneer, Daejeon, Korea) at day 3 after cell seeding. siRNA treatment was three times with 3-day intervals. After the final treatment, 2 hours later, cells were harvested for further analysis.

### 2.7. Flow Cytometric Analysis

PBL and peritoneal macrophages of mice were washed with phosphate-buffered saline (PBS) and stained with PerCP-eFluor-labeled anti-mouse CD40, eFluor 660-labeled anti-mouse CD83, PE-Cyanine7-labeled anti-mouse CD80, and FITC-labeled anti-mouse CD86 (eBioscience, San Diego, CA, USA) at 4°C for 30 min in the dark. For identification of regulatory T cells (Treg cells), isolated PBL was stained with PE-Cyanine7-labeled anti-mouse CD4 and PE-labeled anti-mouse CD25 for 30 min at 4°C in the dark. For intranuclear detection of Foxp3, an anti-mouse Foxp3 staining kit (eBioscience, San Diego, CA, USA) was used, according to the manufacturer's instructions. Briefly, cells were fixed using Fix/Perm buffer and, after washing with 1x permeabilization buffer, were incubated with PE-Cyanine5-labeled anti-mouse Foxp3 Ab for 30 min at 4°C in the dark. Stained cells were analyzed by a FACS Aria III flow cytometer (Becton Dickinson, San Jose, CA, USA) with ≥10,000 gated cells.

### 2.8. Measurement of Cytokine by Enzyme-Linked Immunosorbent Assay (ELISA)

After each mouse was sacrificed, blood was collected from the heart and serum was analyzed using a commercial ELISA kit for the IL-17 level (R&D Systems, Minneapolis, MN, USA). ELISA was conducted according to the manufacturer's instructions. Absorbance values of samples were read at a wavelength of 450 nm using a Bio-Rad model 170-6850 microplate reader (Hercules, CA, USA). ELISA was repeated three times in duplicate wells.

### 2.9. Morphological Observation under Transmission Electron Microscopy (TEM)

Cellular morphological changes were observed under transmission electron microscopy. Cultured DCs were fixed with Karnovsky's fixative solution for 2 h at room temperature and postfixed with osmium tetroxide for 30 min. Fixed cells were washed with cacodylate buffer, dehydrated in graded ethanol, embedded in epon mixture, and incubated at 60°C for 48 h. Epon blocs were sectioned with an ultramicrotome (Reichert-Jung, Bayreuth, Germany), stained with uranium acetate and lead citrate, and then observed under the electron microscope (Zeiss, Oberkochen, Germany).

### 2.10. Statistical Analysis

All data are represented as mean ± SD. Statistical differences between experimental groups were determined by Student's *t*-test and Bonferroni correction. Statistical analysis was conducted using MedCalc® version 9.3.0.0. (MedCalc, Ostend, Belgium). Statistical significance was considered when the *p* value was less than 0.05.

## 3. Result

### 3.1. Frequencies of CD40-, CD83-, CD80-, and CD86-Expressing Cells in Normal, HSV-Infected, BD Normal (BDN), and BD Mice

**Figure 1:**
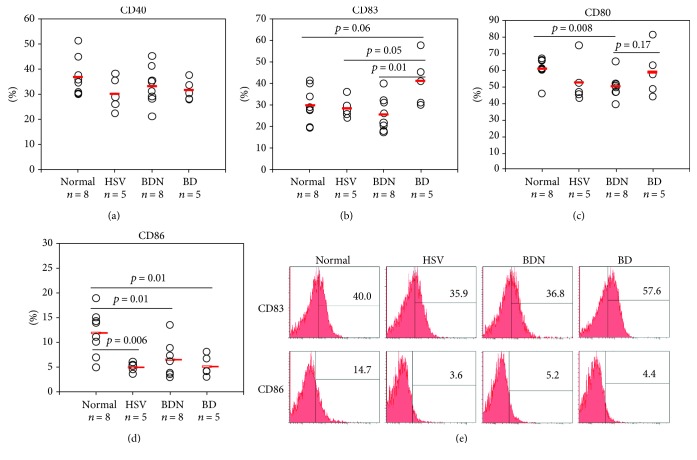
(a–d) Frequencies of DC costimulatory molecules CD40, CD83, CD80, and CD86 in the surface of peripheral blood leukocytes (PBL) were evaluated by flow cytometry analysis. (e) Representative histogram of frequencies of CD83+ and CD86+ cells in PBL. The *p* value was determined by Student's *t*-test. The number of mice used for experiments are 8 in normal, 5 in HSV-1, 8 in BDN, and 5 in BD. *n* indicates the number of mice in each group. Experiments were performed more than three independent times.

Frequencies of DC-expressing costimulatory molecules CD40+, CD83+, CD80+, and CD86+ cells in PBL of mice were analyzed by FACS. Frequencies of CD83+ cells in BD mice (*n* = 5) were significantly elevated compared to those in BDN (*n* = 8) mice (41.0 ± 11.28% vs. 25.85 ± 7.86%, *p* = 0.01), HSV-infected mice (*n* = 5) (28.56 ± 4.59%, *p* = 0.05), and control mice (*n* = 8) (29.92 ± 8.18%, *p* = 0.06) (Figure 1(b)). However, frequencies of CD86+ cells in PBL of BD mice were significantly downregulated compared to those in healthy control mice (5.18 ± 2.11% vs. 11.91 ± 4.55%, *p* = 0.01) (Figure 1(d)). Frequencies of CD86+ cells were also downregulated in HSV-infected mice (4.94 ± 0.92% vs. 11.91 ± 4.55%, *p* = 0.006) and BDN mice (6.47 ± 3.47% vs. 11.91 ± 4.55%, *p* = 0.01) compared with control mice. Frequencies of CD80+ cells in BDN mice were downregulated compared to those in control mice (50.15 ± 7.30% vs. 60.88 ± 6.70%, *p* = 0.008) (Figure 1(c)). However, there was no statistically significant difference in the frequencies of CD40+ cells among groups (Figure 1(a)).  Figure 1(e) shows representative histograms of CD83+ cells and CD86+ cells in normal healthy control, HSV, BDN, and BD mice (*n* indicates the number of mice used in the analysis). To know what population of PBL cells was more correlated to the expression of CD83, the population was gated and CD83+ cells were analyzed. In granulocytes, the frequencies of CD83+ cells were significantly different in BD mice compared with normal (64.74 ± 16.44% vs. 41.86 ± 14.84%, *p* = 0.02) and BDN mice (64.74 ± 16.44% vs. 28.7 ± 5.70%, *p* = 0.0002). In lymphocytes and in monocytes, the frequencies of CD83+ cells were not significantly different between BD and control groups (Fig. [Supplementary-material supplementary-material-1] of the Supplementary Data).

### 3.2. Administration of Abatacept Inhibits CD83+ Cell Frequencies in Normal Mice in a Dose- and Time-Dependent Manner and Improves BD Symptoms

**Figure 2:**
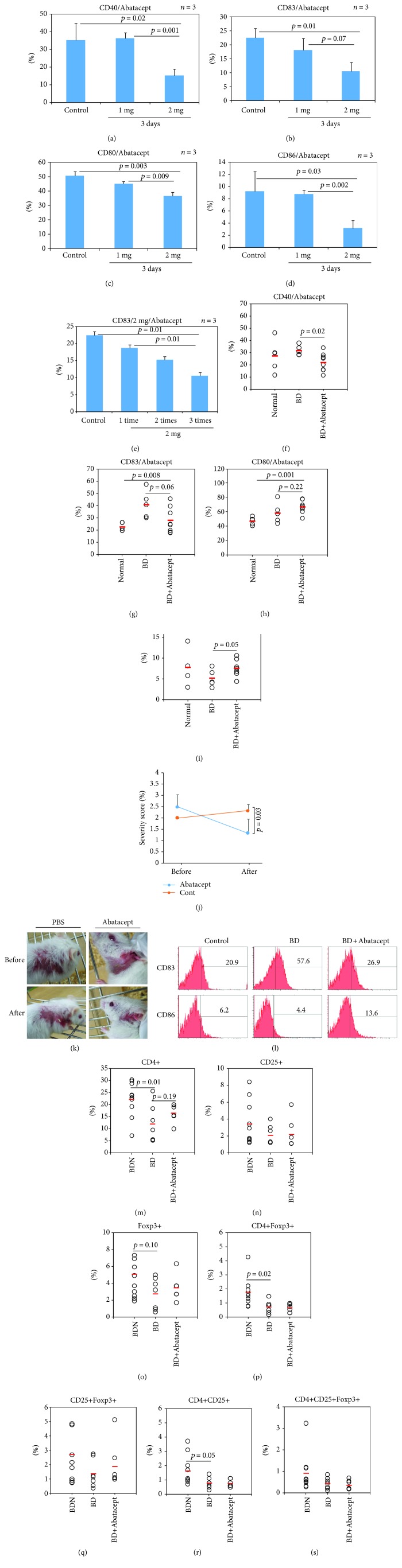
Abatacept affected the frequencies of CD83+ cells in normal and BD mice. (a–d) Frequencies of DC costimulatory molecules CD40, CD83, CD80, and CD86 in normal mice treated with Abatacept in the surface of peripheral blood leukocytes were evaluated by FACS analysis (*n* = 3 for each group). (e) Abatacept 2 mg for 1 to 3 days in normal mice was evaluated by FACS analysis (*n* = 3 for each group). (f–i) Frequencies of CD40, CD83, CD80, and CD86 in BD mice treated with Abatacept were evaluated by FACS analysis (*n* = 5 in normal, *n* = 5 in BD, and *n* = 8 in BD+Abatacept). (j, k) Abatacept treatment decreased the disease severity score and improved symptoms. (l) Representative histograms of CD83+ and CD86+ cell frequencies in PBL of BD mice treated with Abatacept. Regulatory T cells in BD mice were analyzed by FACS analysis. (m–s) Frequencies of CD4+, CD4+Foxp3+, CD4+CD25+, and CD4+CD25+Foxp3+ Treg cells in BDN, BD, and Abatacept-treated BD mice were evaluated by FACS analysis (*n* = 10 in BDN, *n* = 7 in BD, and *n* = 5 in BD+Abatacept). *n* indicates the number of mice used in each group. The *p* value was determined by Student's *t*-test. Experiments were performed more than three independent times.

Abatacept is a fusion protein of the extracellular domain of CTLA-4 and immunoglobulin Fc portion known as a CD80/86 blocker [24]. Abatacept treatment of 2 mg/mouse, once a day for 3 consecutive days, to normal mice significantly decreased frequencies of CD40+ cells (15.26 ± 3.77% vs. 35.13 ± 9.50%, *p* = 0.02), CD83+ cells (10.46 ± 3.25% vs. 22.43 ± 3.44%, *p* = 0.01), CD80+ cells (36.66 ± 2.74% vs. 50.86 ± 2.77%, *p* = 0.003), and CD86+ cells (3.16 ± 1.25% vs. 9.26 ± 3.23%, *p* = 0.03) (Figures 2(a)–2(d)) in PBL of normal mice compared to the control. CD83+ cell frequencies were also decreased after Abatacept treatment in a time-dependent manner from day 1 to day 3 (18.63 ± 1.76% vs. 10.46 ± 3.25%, *p* = 0.01) (Figure 2(e)). GC7 (*N*1-guanyl-1,7-diaminoheptane), an inhibitor of hypusine formation, also known as an inhibitor of CD83 [15], was used to treat normal mice to determine whether GC7 could decrease CD83+ cell frequencies. The results showed that there was no significant difference in CD83+ cell frequencies between before and after GC7 treatment (Fig. [Supplementary-material supplementary-material-1] of the Supplementary Data). Frequencies of CD40+ cells (21.91 ± 7.58% vs. 31.64 ± 4.11%, *p* = 0.02) and CD83+ cells (28.08 ± 10.54% vs. 41.0 ± 11.28%, *p* = 0.06) were significantly decreased in BD mice treated with Abatacept compared to those in nontreated BD mice (Figures 2(f) and 2(g)). However, frequencies of CD86+ cells (7.62 ± 0.93% vs. 5.18 ± 2.11%, *p* = 0.05) were increased in BD mice after treatment with Abatacept compared to those in non-treated control BD mice (Figure 2(i)). Frequencies of CD80+ cells (67.16 ± 8.65% vs. 58.92 ± 14.53%, *p* = 0.22) were not significantly changed after treatment with Abatacept (Figure 2(h)).

### 3.3. Frequencies of Regulatory T Cells in Abatacept-Treated BD Mice

CD4+CD25+ Foxp3+ regulatory T (Treg) cells were analyzed by flow cytometry analysis. Frequencies of CD4+ T cell in BD mice were significantly downregulated compared to those in BDN mice (11.92 ± 7.78% vs. 22.23 ± 7.16%, *p* = 0.01). They were slightly elevated after Abatacept treatment (before and after treatment: 11.92 ± 7.78% vs. 16.41 ± 3.54%, *p* = 0.19) (Figure 2(m)). Frequencies of Foxp3+ cells were more downregulated in BD mice than those in BDN mice (2.76 ± 1.86% vs. 5.06 ± 3.20%, *p* = 0.10). They were marginally but not significantly increased by Abatacept treatment (before and after treatment: 2.76 ± 1.86% vs. 3.50 ± 1.44%, *p* = 0.42) (Figure 2(o)). Frequencies of CD4+Foxp3+ cells were significantly decreased in BD mice compared to those in BDN mice (0.71 ± 0.43% vs. 1.77 ± 1.01%, *p* = 0.02) (Figure 2(p)). Frequencies of CD4+CD25+ cells were also lower in BD mice than those in BDN mice (0.78 ± 0.37% vs. 1.62 ± 1.02%, *p* = 0.05) (Figure 2(r)). Frequencies of CD4+CD25+ Foxp3+ Treg cells were downregulated in BD mice compared to BDN mice. However, such difference was not statistically significant (Figure 2(s)).

### 3.4. Abatacept Treatment Decreases the Disease Severity Score and Ameliorates BD Symptoms in Mice

To determine whether Abatacept could manage BD symptoms, 2 mg Abatacept was intraperitoneally injected to BD mice 3 times with 2-day intervals and BD symptoms were traced for one week. Only PBS was injected to BD mice as control. The severity score of Abatacept-treated BD mice was significantly decreased at one week after treatment compared to that of PBS-treated BD mice (1.33 ± 0.62% vs. 2.33 ± 0.28%, *p* = 0.03) (Figure 2(j)).  Figure 2(k) shows the changes of BD symptoms at 1 week after Abatacept treatment.  Figure 2(l) shows representative histograms of CD83+ and CD86+ cells in normal, BD, and Abatacept-treated BD mice.

### 3.5. CD83 siRNA Suppresses CD83+ Cell Frequencies in Normal Mice

**Figure 3:**
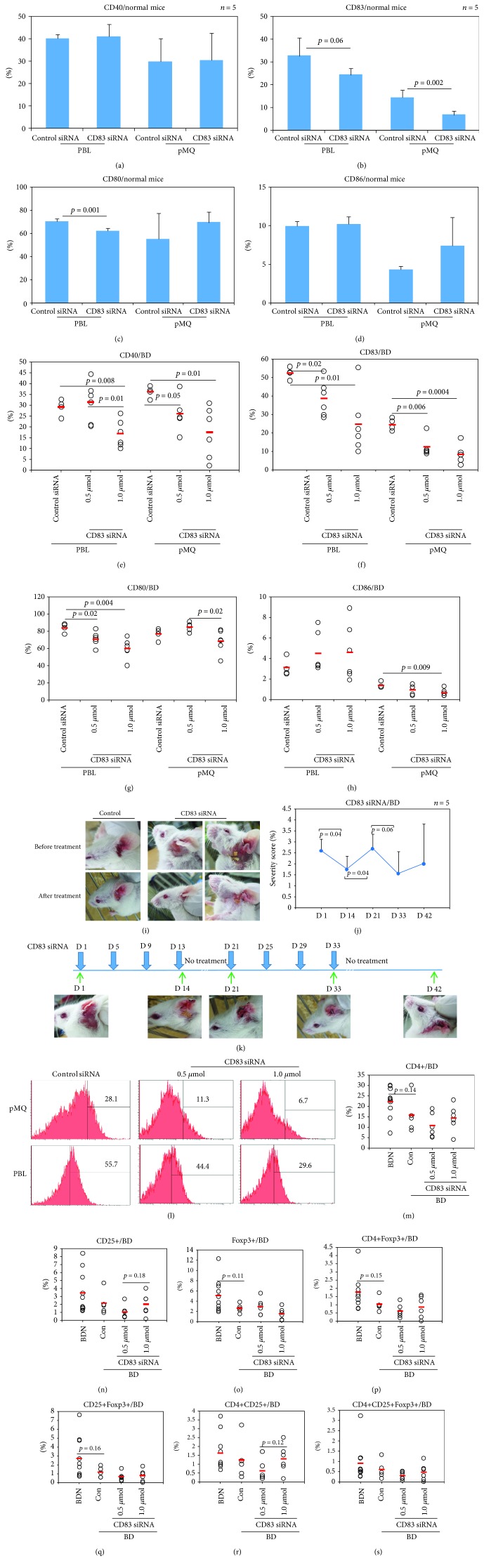
CD83 siRNA decreases the frequencies of CD83+ cells in normal mice. (a–d) Frequencies of CD40, CD83, CD80, and CD86 in the surface of peripheral blood leukocytes (PBL) and in the peritoneal macrophages of normal mice treated with CD83 siRNA were evaluated by FACS analysis (*n* = 5 in each group). (e–h) CD83 siRNA decreases frequencies of CD83 in BD mice. Frequencies of CD40, CD83, CD80, and CD86 in the surface of peripheral blood leukocytes (PBL) and in the peritoneal macrophage of BD mice treated with CD83 siRNA were evaluated by flow cytometry analysis. *n* = 4, BD symptomatic mice were used in control groups as scramble siRNA; *n* = 6, BD symptomatic mice were used in the 0.5 *μ*mol and 1.0 *μ*mol CD83 siRNA-treated groups. (i, j) CD83 siRNA treatment significantly decreased the severity score of BD mice and improved symptoms. (k) Treatment schedule of CD83 siRNA to BD mice. (l) Representative histogram of CD83+ cell frequencies in PBL and peritoneal macrophage of BD mice treated with CD83 siRNA. (m–s) Regulatory T cells in BD mice analyzed by FACS analysis. Frequencies of CD4+, CD4+Foxp3+, CD4+CD25+, and CD4+CD25+Foxp3+ regulatory T cells in BDN (*n* = 10), BD (*n* = 5), jetPEI-treated BD (*n* = 6), and CD83 siRNA-treated BD mice (*n* = 6) were evaluated by FACS analysis. *n* indicates the number of mice used in each group. The *p* value was determined by Student's *t*-test. Experiments were performed more than three independent times.

CD83 siRNA was used to treat normal mice to suppress the surface expression of CD83. Frequencies of CD83+ cells were measured by FACS analysis. Intraperitoneal treatment of CD83 siRNA to normal mice decreased frequencies of CD83+ cells in peritoneal macrophages (6.74 ± 1.62% vs. 14.4 ± 3.12%, *p* = 0.002) and in PBL (24.3 ± 3.01% vs. 32.6 ± 7.83%, *p* = 0.06) (Figure 3(b)) compared with the scramble siRNA treatment group. CD83 siRNA treatment also decreased CD80+ cell frequencies in PBL (62.18 ± 2.40% vs. 70.0 ± 2.35%, *p* = 0.001) compared with the scramble siRNA treatment group (Figure 3(c)). There were no significant differences observed in CD40+ and CD86+ cells in PBL and peritoneal macrophages.

### 3.6. CD83 siRNA Treatment Affects BD Symptoms and Decreases the Disease Severity Score of Mice

The frequencies of CD83+ cells in BD mice treated with CD83 siRNA were measured by FACS analysis. Intraperitoneal injection of CD83 siRNA at 0.5 *μ*mol/mouse (12.32 ± 5.67% vs. 24.5 ± 3.19%, *p* = 0.006) and 1 *μ*mol/mouse (8.38 ± 4.95% vs. 24.5 ± 3.19%, *p* = 0.0004) to BD mice significantly decreased the frequencies of CD83+ cells in peritoneal macrophages, compared with injection with scramble siRNA (Figure 3(f)). In PBL, the 0.5 *μ*mol- (38.51 ± 9.69% vs. 52.22 ± 3.07%, *p* = 0.02) and 1 *μ*mol-treated groups (24.66 ± 16.52% vs. 52.22 ± 3.07%, *p* = 0.01) showed lower frequencies of CD83+ cells compared to the scramble siRNA-treated group (Figure 3(f)). Frequencies of CD40+ cells in peritoneal macrophages were downregulated in 0.5 *μ*mol (25.96 ± 8.44% vs 36.07 ± 2.67%, *p* = 0.05) and 1 *μ*mol (17.38 ± 11.80% vs 36.07 ± 2.67%, *p* = 0.01) CD83 siRNA-treated BD mice compared to those in the scramble siRNA-treated control group (Figure 3(e)). They were also decreased in PBL of the 1 *μ*mol CD83 siRNA-treated group compared to those in the scramble siRNA-treated control (16.85 ± 6.30% vs. 29.1 ± 3.77%, *p* = 0.008) (Figure 3(e)). Frequencies of CD86+ cells in peritoneal macrophages of 1 *μ*mol CD83 siRNA-treated BD mice were also decreased compared to those in the scramble siRNA-treated control group (0.65 ± 0.35% vs. 1.37 ± 0.28%, *p* = 0.009), although no significant differences were found in PBL (Figure 3(h)). Frequencies of CD80+ cells in PBL in the 0.5 *μ*mol (71.15 ± 8.23% vs. 84.1 ± 5.29%, *p* = 0.02) and in the 1 *μ*mol (59.96 ± 11.48% vs. 84.1 ± 5.29%, *p* = 0.004) CD83 siRNA-treated groups also showed downregulation compared to those in the scramble siRNA-treated control group (Figure 3(g)). In peritoneal macrophages, the 1 *μ*mol-treated group showed lower frequencies of CD80+ cells compared to the 0.5 *μ*mol-treated group (68.4 ± 13.26% vs. 84.94 ± 5.09%, *p* = 0.02) (Figure 3(g)). CD83 siRNA-treated BD symptomatic mice showed improved symptoms (Figure 3(i)). The disease severity score was also significantly decreased after 2 weeks (1.75 ± 0.61 vs. 2.60 ± 0.54, *p* = 0.04) (Figure 3(j)). Discontinuation of treatment increased the disease severity score and deteriorated symptoms. Retreatment brought improvement again and decreased the disease severity score (Figure 3(j)).  Figure 3(k) shows changes of BD symptoms after CD83 siRNA treatment to mice and time intervals.  Figure 3(l) shows representative histograms of CD83+ cells in BD mice treated with CD83 siRNA.

### 3.7. Frequencies of Regulatory T Cells in CD83 siRNA-Treated BD Mice

Frequencies of CD4+Foxp3+, CD25+Foxp3+, CD4+CD25+, and CD4+CD25+Foxp3+ Treg cells were analyzed by FACS. Frequencies of CD4+ T cells in BD mice were downregulated compared to those in BDN mice (15.76 ± 8.59% vs. 22.23 ± 7.16%, *p* = 0.14) (Figure 3(m)). Frequencies of CD4+Foxp3+ and CD25+Foxp3+ cells were slightly decreased in BD mice compared to those in BDN mice (CD4+Foxp3+: 1.04 ± 0.39% vs. 1.77 ± 1.01%, *p* = 0.15; CD25+Foxp3+: 1.15 ± 0.58% vs. 2.71 ± 2.31%, *p* = 0.16) (Figures 3(p) and 3(q)). Frequencies of CD4+CD25+Foxp3+ Treg cells were similar between 1 *μ*mole CD83 siRNA-treated BD mice and BD control (0.49 ± 0.41% vs. 0.61 ± 0.44%, *p* = 0.64) (Figure 3(s)).

### 3.8. CD83 siRNA Treatment Downregulates IL-17 Levels in the Plasma of BD Mice

**Figure 4:**
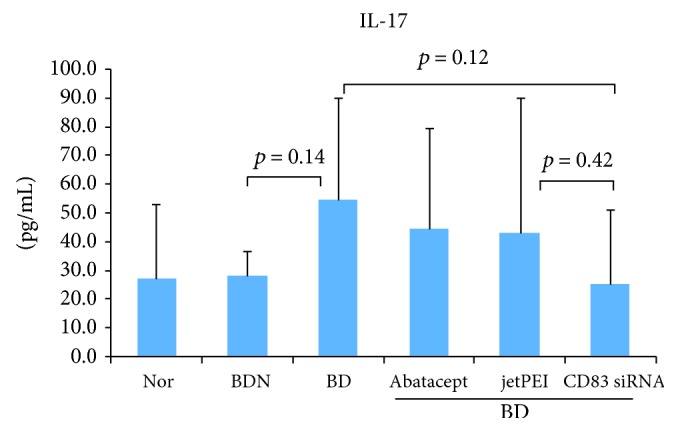
Serum interleukin 17 (IL-17) levels in BD mice after being treated with CD83 siRNA were analyzed by ELISA. Data represent two independent experiments.

Several studies of BD have demonstrated that a significant increase of serum IL-17 is an indicator of reactive or recurrence of infection [25, 26]. To determine whether CD83 siRNA could improve the symptoms of BD by downregulating IL-17, plasma IL-17 levels were measured in CD83 siRNA-treated BD mice by ELISA. The IL-17 level was downregulated in CD83 siRNA-treated BD symptomatic mice (25.2 ± 26.2 pg/mL) compared to that in nontreated BD mice (54.4 ± 35.3 pg/mL) (*p* = 0.12) (Figure 4). This result suggests that the inhibition of CD83 has a protective role against BD.

### 3.9. CD83 siRNA Suppresses CD83+ Cell Frequencies and Modulates the Morphology of Dendrites in In Vitro-Cultured Dendritic Cells

**Figure 5:**
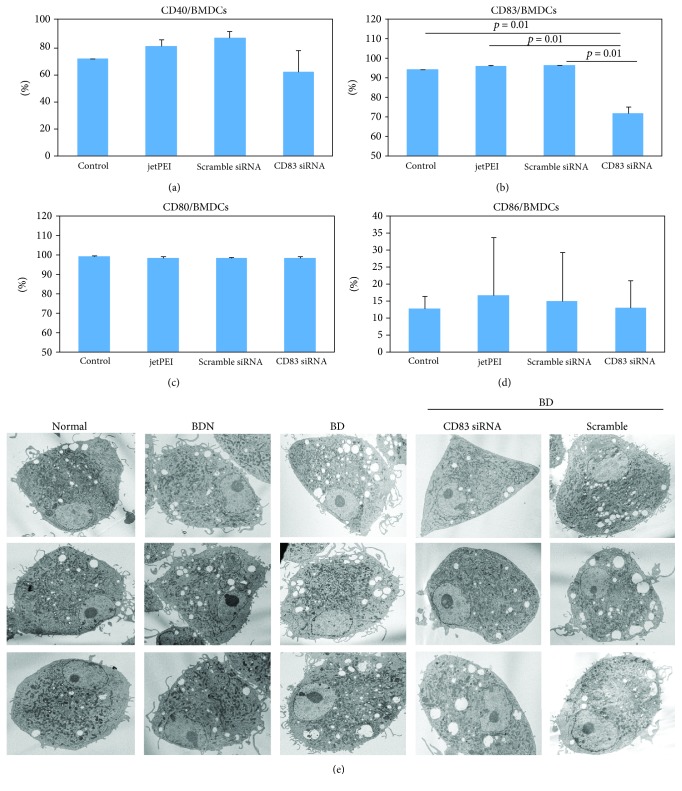
Bone marrow-derived DCs were cultured and treated with CD83 siRNA. (a–d) Frequencies of CD40-, CD83-, CD80-, and CD86-positive cells were measured by FACS analysis. (e) Morphological changes were observed under a transmission electron microscope. Data represent two independent experiments.

CD83 siRNA was used to treat bone marrow-derived dendritic cells to elucidate whether CD83 siRNA can suppress CD83 in *in vitro* dendritic cell cultures. Frequencies of CD83+ cells were measured by FACS analysis. CD83 siRNA significantly decreased the frequencies of CD83+ cells compared with the scramble siRNA treatment group (71.7 ± 3.53% vs. 96.3 ± 0.14%, *p* = 0.01) (Figure 5(b)). There were no significant differences observed among the groups in other costimulatory molecules (Figures 5(a), 5(c), and 5(d)). By transmission electron microscopy, dendrites of the plasma membrane in cultured dendritic cells were shown to be decreased in the CD83 siRNA-treated group when compared to those in the nontreated BD or scramble siRNA-treated BD group (Figure 5(e)). The morphology of cytoplasmic organelles was not different between the CD83 siRNA- and scramble siRNA-treated groups, except for vacuoles; CD83 siRNA-treated dendritic cells showed less vacuoles than scramble siRNA-treated cells.

## 4. Discussion

DCs are primary initiators and frontline cells of immune response. They are involved in the interface between innate immunity and adaptive immunity. DCs contribute to both central immunity and peripheral immunity [27]. Signals of costimulatory molecules such as CD40, CD80, CD83, and CD86 can regulate the maturation of DCs [28]. Mitogen-activated protein kinase [29], signal transducers, and activators of transcription [30] are also involved in the maturation of DCs. Costimulatory molecules are receptors/ligands that can regulate inflammation [31]. Among those costimulatory molecules for DC maturation, CD83 is a functional molecule in the interplay between DCs and lymphocytes. CD83 is expressed as membrane-bound and soluble form (sCD83) [32]. Expression of the cell surface CD83 is upregulated upon DC activation. It is primarily used to identify the maturation or activation of DCs [33]. A significant number of CD83-expressing DCs have been observed in patients with Crohn's disease [34]. Treatment with soluble CD83 can inhibit pathological symptoms of experimental autoimmune encephalomyelitis (EAE) [35] and experimental autoimmune uveitis (EAU) [36]. In the present study, frequencies of CD83-expressing cells in the PBL surface were upregulated in BD symptomatic mice. This suggests that the elevated level of CD83 plays a pathogenic role in BD.

CD80 and CD86, also known as DC activation molecules, are stimulated via CD28 on the T cell surface. They provide T cell activation signals [37, 38]. Upregulation of CD80 and loss of constitutive properties of CD86 have been associated with the severity of disease and inflammation in humans [31]. Costimulatory interactions of CD80 and CD86 to T cells are required for the activation of autoreactive T cells and induction of arthritis [39]. Our investigation showed that in symptomatic BD mice, the proportion of CD80 was slightly increased, while the proportion of CD86 was decreased in symptomatic BD mice. It has been reported that the DC costimulatory molecule CD40 can bind to its ligand CD40L which is transiently expressed on T cells under inflammatory conditions and expressed significantly greater in ulcerative colitis and Crohn's disease [40]. We found that in BD symptomatic mice, frequencies of CD40 expressing cells were not significantly elevated.

GC7 (*N*1-guanyl-1,7-diaminoheptane) is an inhibitor of CD83 that interferes with CD83 surface expression and inhibits DC-mediated T cell activation by affecting the nuclear cytoplasmic translocation of CD83 mRNA [15]. However, in our study, GC7 treatment in normal mice did not show any significant difference in CD83 inhibition.

Abatacept, a recombinant fusion protein of the extracellular domain of CTLA-4 and the Fc region of human IgG1, can selectively modulate costimulatory signals CD80/CD86-CD28 for T cell activation [41, 42]. Abatacept significantly decreased CD83+ cells in a dose- and time-dependent manner in the present study. It also downregulated proportions of CD40, CD80, and CD86 in normal mice. Abatacept has been approved for use in patients with highly active rheumatoid arthritis (RA). It can improve the symptoms of RA and decrease disease activity and progression of structural damage [41, 42]. Abatacept treatment to the systemic sclerosis mouse model is effective in preventing fibrosis [43]. In our study, treatment of symptomatic BD mice with Abatacept significantly reduced the frequencies of CD83+ and CD40+ cells. We also found that Abatacept treatment to BD symptomatic mice decreased the disease severity and improved symptoms. Treg cells play an important role in the suppression of inflammation in autoimmune diseases. Patients with rheumatoid arthritis treated with Abatacept show increased IL-10 by producing CD4+CD25-LAG3+ Treg cells [44]. Abatacept can reduce T cell apoptosis and upregulate the proportion of Treg cells in RA patients [45]. Increased CD4+CD25+ Treg cells are associated with improved inflammatory symptoms in BD mice [46]. In the present study, Abatacept treatment to BD mice increased the frequencies of CD4+ T cells and CD25+Foxp3+ cells compared to nontreated control. This suggests that DC costimulatory molecules are associated with BD symptoms.

It has been suggested that the inhibition of CD83 mRNA transport can be applied to develop therapeutics for autoimmune diseases [16, 47]. Preventing cell surface expression of CD83 can significantly inhibit DC-mediated T cell activation [15]. siRNA is considered to be a potent drug molecule that can silence genes associated with pathogenesis, especially in the treatment of inflammatory diseases [48, 49]. The siRNA binds to the RNA-induced silencing complex (RISC); then the passenger siRNA chain departs and initiates the process of RNA interference process, causing mRNA fragmentation and degradation [50, 51]. Injection of CD83 siRNA into normal mice significantly reduced the proportion of CD83-expressing cells in PBL and peritoneal macrophages. In addition, the frequencies of CD83+ cells in peritoneal macrophages and PBL of BD symptomatic mice treated with CD83 siRNA were significantly reduced. We also found that BD mice treated with CD83 siRNA showed reduced frequencies of CD40+ cells in peritoneal macrophages and PBL and decreased frequencies of CD80+ and CD86+ cells in peritoneal macrophages. CD4+CD25+ Treg cells can maintain self-tolerance and suppress autoimmune response and upregulation of CD4+CD25+ Treg cells in BD mice that are associated with disease improvement [46]. BD mice treated with CD83 siRNA showed a significantly reduced severity score of the disease, with improved symptoms. However, discontinuation of CD83 siRNA treatment showed deterioration of symptoms that were again improved after CD83 siRNA treatment. This clearly suggests that CD83 is a potential molecule for modulating BD symptoms. Upon activation of DCs, immature DCs can migrate to the draining lymph node and become mature antigen-presenting cells [52]. During maturation, DCs can change the surface expression of costimulatory molecules and morphology, including the expansion of dendrites and increase of lysosomes [53]. In our *in vitro* study, cultured bone marrow-derived DCs showed enhancement of dendrites in normal, BDN, and BD mice while treatment with CD83 siRNA showed less dendrite compared to nontreated BD and scramble siRNA-treated DCs. This provides evidence that CD83 plays a potent role in the maturation of DCs.

Th17 cells play an important role in autoimmunity. Expression of cytokine IL-17 is a special characteristic of Th17 cells. Increased production of IL-17 has been associated with several inflammatory disorders such as rheumatoid arthritis (RA), ankylosing spondylitis (AS), and Behçet's disease (BD) [54–56]. A significantly higher level of IL-17 has been observed in active BD patients [57, 58]. Downregulation of IL-17 is associated with the reduction of symptoms in BD mice when treated with miRNA21, IL-6 siRNA, recombinant IL-4, and N-acetyl-d-galactosamine 4-sulfate [49]. In our study, BD symptomatic mice treated with CD83 siRNA showed improved BD symptoms and downregulation of IL-17 in serum.

## 5. Conclusion

In summary, a high proportion of CD83+ cells in BD mice is correlated with BD symptoms. Inhibition of CD83 by treatment with CD83 siRNA to BD mice can significantly reduce the proportions of CD83+, and that is associated with disease improvement. Discontinuation and retreatment of CD83 siRNA brought changes of symptoms. According to these data, it is clear that CD83 plays an important role in modulating BD symptoms. Our results suggest that targeting CD83 molecules can be used as a strategy to develop therapy for BD management.

## Data Availability

The data used to support the findings of this study are available from the corresponding author upon request.

## Supplementary Materials

Supplementary Materials Supplementary Figure S1: (A-C) frequencies of CD83+ cells in granulocytes, lymphocytes, and monocytes of normal (*n* = 8), HSV-1 (*n* = 5), BDN (*n* = 8), and BD mice (*n* = 5) were evaluated by FACS analysis (A-C). (D) Representative histogram of CD83+ cells in granulocytes. *n* indicates the number of mice used in each group. The *p* value was determined by Student's *t*-test. Supplementary Figure S2: (A-D) GC7 (*N*1-guanyl-1,7-diaminoheptane) was used to treat normal mice, and frequencies of CD40, CD83, CD80, and CD86 expressing cells in the PBL surface were evaluated by FACS analysis (*n* = 3 in each group).
