# Supplementary material for: Inhibition of CD83 Alleviates Systemic Inflammation in Herpes Simplex Virus Type 1-Induced Behçet's Disease Model Mouse
Source: Mediators Inflamm. 2019 Sep 9;2019:5761392. doi: 10.1155/2019/5761392 (PMC6754941; doi:10.1155/2019/5761392)
Supplement: Supplementary Materials — Supplementary Figure S1: (A-C) frequencies of CD83+ cells in granulocytes, lymphocytes, and monocytes of normal (n = 8), HSV-1 (n = 5), BDN (n = 8), and BD mice (n = 5) were evaluated by FACS analysis (A-C). (D) Representative histogram of CD83+ cells in granulocytes. n indicates the number of mice used in each group. The p value was determined by Student's t-test. Supplementary Figure S2: (A-D) GC7 (N1-guanyl-1,7-diaminoheptane) was used to treat normal mice, and frequencies of CD40, CD83, CD80, and CD86 expressing cells in the PBL surface were evaluated by FACS analysis (n = 3 in each group). [file 5761392.f1.pdf]

Supplementary Data

Supplementary Figure S1.

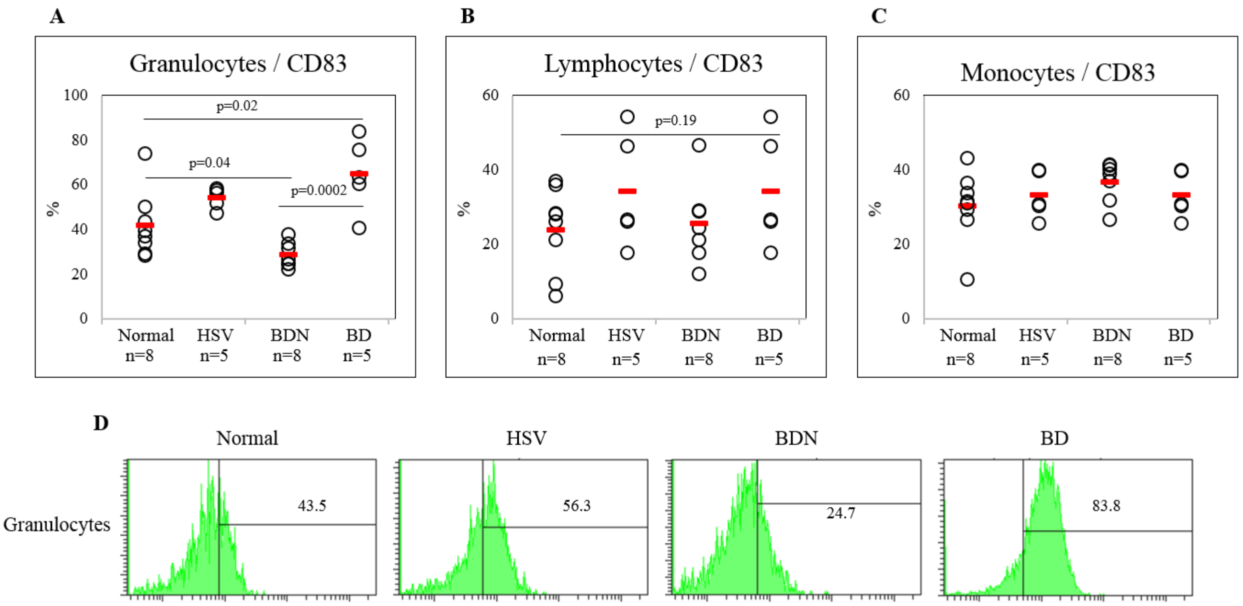

Supplementary Figure 1. (A-C) Frequencies of CD83+ cells in granulocytes, lymphocytes and monocytes of normal (n=8), HSV-1 (n=5), BDN (n=8), and BD mice (n=5) were evaluated by FACS analysis (A-C). (D) Representative histogram of CD83+ cells in granulocytes. n indicates the number of mice used in each group. The *p*-value was determined by Student’s *t*-test.

Supplementary Figure S2.

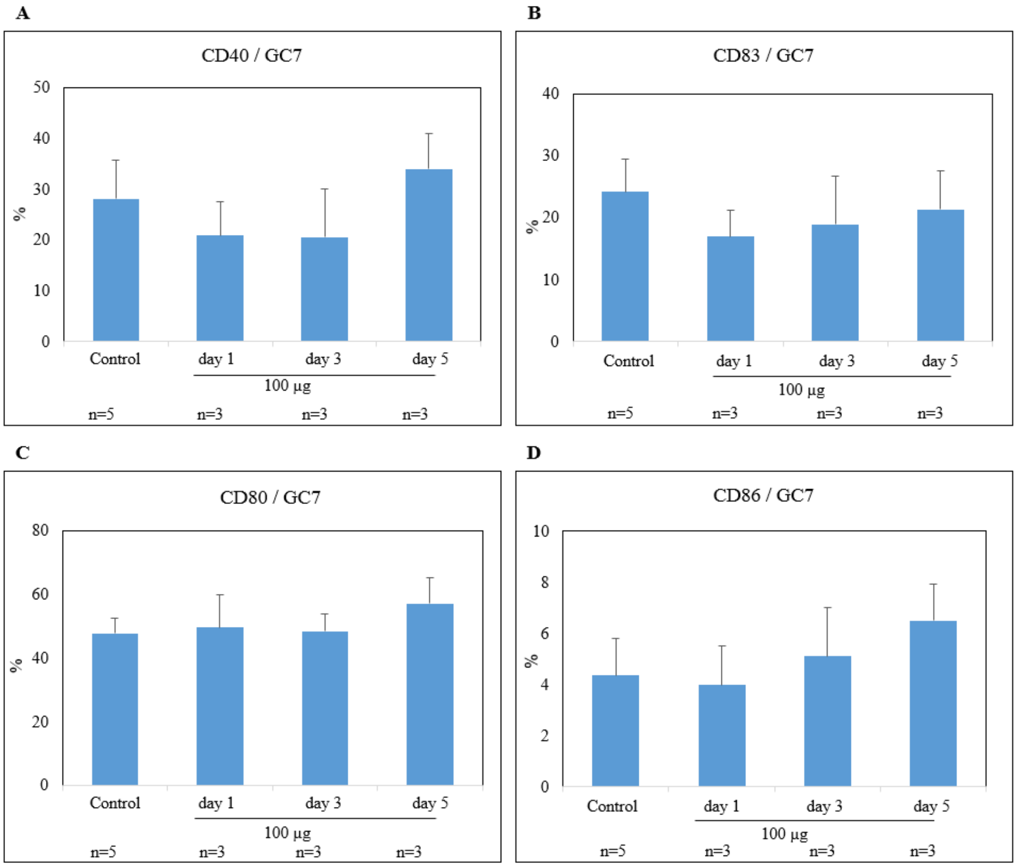

Supplementary Figure S2. (A-D) GC7 (*N* 1-guanyl-1,7-diaminoheptane) was used to treat normal mice, and frequencies of CD40, CD83, CD80, and CD86 expressing cells in PBL surface were evaluated by FACS analysis (n=3 in each group).
